# Targeting RTK-PI3K-mTOR Axis in Gliomas: An Update

**DOI:** 10.3390/ijms22094899

**Published:** 2021-05-05

**Authors:** Mayra Colardo, Marco Segatto, Sabrina Di Bartolomeo

**Affiliations:** Department of Biosciences and Territory, University of Molise, 86090 Pesche， IS, Italy; m.colardo@studenti.unimol.it (M.C.); marco.segatto@unimol.it (M.S.)

**Keywords:** RTKs, EGFR, PI3K-mTOR, glioma, HCGs

## Abstract

Gliomas are the most common and challenging malignancies of the central nervous system (CNS), due to their infiltrative nature, tendency to recurrence, and poor response to treatments. Indeed, despite the advances in neurosurgical techniques and in radiation therapy, the modest effects of therapy are still challenging. Moreover, tumor recurrence is associated with the onset of therapy resistance; it is therefore critical to identify effective and well-tolerated pharmacological approaches capable of inducing durable responses in the appropriate patient groups. Molecular alterations of the RTK/PI3K/Akt/mTOR signaling pathway are typical hallmarks of glioma, and several clinical trials targeting one or more players of this axis have been launched, showing disappointing results so far, due to the scarce BBB permeability of certain compounds or to the occurrence of resistance/tolerance mechanisms. However, as RTK/PI3K/mTOR is one of the pivotal pathways regulating cell growth and survival in cancer biology, targeting still remains a strong rationale for developing strategies against gliomas. Future rigorous clinical studies, aimed at addressing the tumor heterogeneity, the interaction with the microenvironment, as well as diverse posology adjustments, are needed—which might unravel the therapeutic efficacy and response prediction of an RTK/PI3K/mTOR-based approach.

## 1. Introduction

Gliomas are the most common and malignant type of primary tumors of the central nervous system (CNS) [1]. According to the 2007 report of World Health Organization (WHO) classification, malignant gliomas include grade II (Low-grade gliomas, LGGs), grade III, and grade IV (HGGs). HGGs include anaplastic astrocytoma (AA), anaplastic oligodendroglioma (AO), and glioblastoma (GBM) and present the worst prognosis and a high mortality rate [1]. Although the 2007 classification is still adopted by clinicians, a new classification was introduced in 2016 by WHO, mainly based on genetic and molecular abnormalities, but also including clinicopathological features [2]. In addition to mutations in the gene encoding for isocitrate dehydrogenase enzyme 1/2 (IDH1/2), which identify different GBM subgroups, genetically altered RTKs and their downstream effectors are likely the most abundant targetable driver mutations in GBM. In particular, the Cancer Genome Atlas project (TCGA) reported a significant alteration in three core signaling pathways, including RTK/RAS/PI3K (88%), p53 (87%), and retinoblastoma protein (78%) in the collected samples from patients with primary GBM [3]. This is also consistent with mouse models of GBM, in which the reconstitution of the genetically altered components of growth factor signaling pathways, recreates histologically identical GBMs [4,5]. EGFR alterations are the dominant, but not the exclusive, RTK abnormalities observed in primary GBMs; sometimes, these are the sole RTK lesions, and noteworthy, usually occur in the context of other PI3K-pathway activating alterations and in the presence of CDKN2A loss and inactivation. Amplification of EGFR (45%), gain of function in PIK3CA (15%), or loss of PTEN (36%) activate the lipid kinase PI3K and its downstream target, the pleckstrin-homology-domain serine-threonine kinase Akt, also known as Protein kinase B (PKB), which regulates over 40 downstream targets, one of which is mammalian target of Rapamycin (mTOR) kinase [6]. mTOR functions in two distinct multi-protein complexes, namely, mTOR complex 1 (mTORC1), defined by association with the regulatory associated protein RAPTOR, and mTOR complex 2 (mTORC2), defined by the association with the rapamycin-insensitive companion RICTOR [7]. mTOR kinase is considered the cellular nutrient sensor, as mTORC1 regulates cell size and growth in response to nutrients levels, and mTORC2 regulates both the cytoskeleton and Akt activation [7].

Patients with HGG usually require surgical resection combined with radiotherapy or/and temozolomide (TMZ) chemotherapy [8]. However, the average survival time for these patients is of only 14,4 months, likely due to resistance mechanisms. Therefore, it is urgent to identify new therapeutic targets and strategies to improve both survival and lifestyle, which is also significantly compromised. Although preclinical and initial clinical studies inhibiting EGFR and other RTKs, as well as PI3K and mTOR, have only shown modest efficacy in GBM so far [9,10], RTKs and downstream signaling components represent attractive targets for developing novel therapeutic strategies. A further understanding of the molecular and genetic abnormalities in GBM may lead to improved therapies using single agents or combination protocols, enabling these pathways to be effectively targeted in patients.

## 2. RTK Expression and Signaling in Glioma

Receptor tyrosine kinases (RTKs) are a family of cell-surface receptors which regulate critical cellular processes, including proliferation, differentiation, migration, metabolism, and cell cycle, among others [11]. 58 RTKs, divided into 20 families, have been identified in mammals so far [12]. All of them have an extracellular ligand-binding domain, a transmembrane helix, and a cytoplasmic region that has tyrosine kinase activity [12]. Ligand binding to the extracellular domain induces receptor oligomerization, usually dimerization, and juxtaposition of the tyrosine kinase domains of both receptors stabilize the kinase active state [13]. Once activated, each monomer phosphorylates the tyrosine residues located in the cytoplasmic tail of the other monomer, which are subsequently recognized by intracellular proteins containing Src homology-2 (SH2) or phosphotyrosine-binding (PTB) domains, inducing multiple signaling cascades [14]. RTKs de-regulation, due to genetic alteration, abnormal expression, and/or cellular distribution, has been largely involved in the development of several tumors, including gliomas [15]. In particular, the overactivation of EGFR signaling promotes several hallmarks of tumor onset and progression, such as abnormal proliferation and motility, angiogenesis, and apoptosis inhibition [16]. Strikingly, alterations of RTKs can also result in drug resistance [17].

RTK amplifications and/or mutations occur in 66% of the primary GBM samples tested in the Pan Cancer Project TCGA. The amplifications and/or mutations in EGFR were the only RTK lesions observed in 50% of all de novo primary GBMs. Conversely, EGFR genetic alterations only coexist with other RTK lesions in a small proportion (7%) of tumors. EGFR is commonly de-regulated or mutated in GBM, leading to mys-regulation of downstream signaling pathways, that include PI3K, Akt, MAPK, and PLCz. The *EGFR* gene is amplified in more than 50% tumors, and among these, about 40% show rearrangements, mostly resulting in the EGFRvIII variant, a mutant protein with a ligand-independent activity that lacks the ligand domain [3,18]. The EGFR receptor family includes other members: Erb2, ErbB3, and ErbB4, with ErbB2 (Her2/Neu) mutation observed in 8% GBM tested in a TCGA study [19].

In addition to EGFR genetic alterations, amplification/mutation in platelet-derived growth factor receptor (PDGFR), c-Met, Tie, Axl, discoidin domain receptor 1 (DDR1), erythropoietin-producing human hepatocellular carcinoma (Eph) also play a role in GBM biology [20]. Amplification of platelet-derived growth factor receptor alpha polypeptide (PDGFRA) occurs in 13% of GBMs, whereas MET amplifications and fibroblast growth factor receptor (FGFR) mutations, including fusion genes, occur in about 2% of the GBMs [21]. RTKs alterations often co-occur with mutations or deletions in genes encoding for downstream signaling components, such as PI3K or PTEN, or with mutation/deletion of cyclin-dependent kinase inhibitor 2A (CDKN2A) [22]. The cooperation of multiple core pathways for tumor formation has been confirmed in genetically engineered mouse models of GBM [23].

Besides genetic and chromosomal alterations, ligand and/or receptor overexpression are frequent events in cancers, including GBM, and many of them have been associated with increased malignancy and worse patient outcomes [15]. Oncogenic RTKs activation is also responsible for an altered behavior of the cells belonging to the tumor microenvironment (TME), such as endothelial, immune cells, glioma stem cells (GCSs), and others [24]. For instance, it has been established that glioma cells secrete RTKs ligands, which in turn activate receptors in TME cells, thus promoting tumoral transformation.

Furthermore, it has been demonstrated that the expression levels of some RTKs are altered after chemotherapy in patients affected by glioma. RNA sequencing interrogation from 135 primary and 47 recurrent high-grade gliomas have shown that the expression of at least six RTKs genes, among 58 analyzed, appeared to be modified by standardized radio- and chemotherapy [17]. As alterations of RTKs function can increase or reduce the response to therapy, further investigation of RTKs expression profile during patient treatment would be useful to improve clinical risk stratification [25,26,27].

## 3. PI3K/mTOR Axis in Glioma

**Figure 1:**
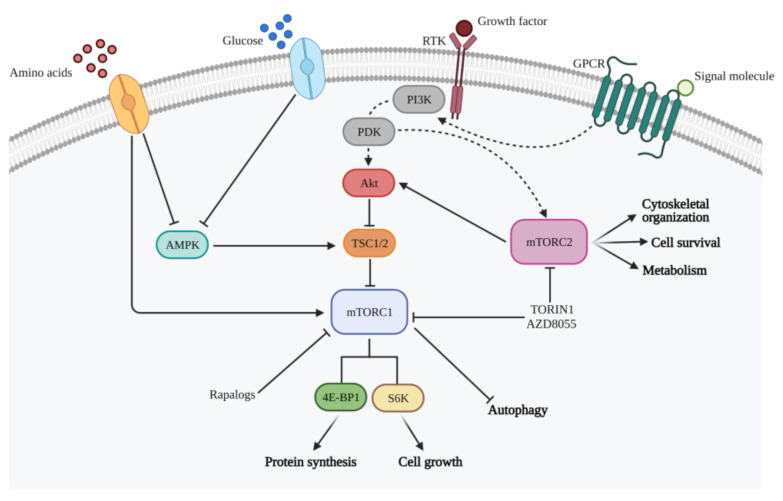
Representative image of the main signaling pathways that regulate mTOR. mTORC1 is able to capture and transduce signals from different stimuli, such as the presence of growth factors, amino acids, and glucose through the mechanisms indicated. Once activated, mTORC1 promotes cell growth and proliferation, inducing various anabolic processes, including protein synthesis, and inhibiting catabolic processes, such as autophagy. mTORC2, on the other hand, performs an important regulatory function of the cytoskeleton, cell survival, and metabolism and phosphorylates the Akt kinase, determining its activation. This image was created with BioRender software.

PI3K/mTOR signaling pathway is involved in crucial cellular functions comprising cell metabolism, growth, survival, and motility [28,29] (Figure 1). PI3K belongs to a family of lipid kinases activated by a large number of RTKs to produce phosphatidylinositol-3,4,5-trisphosphate (PIP3) [30]. PI3Ks comprise three classes: Classes I, divided into IA and IB, II and III [31,32]. The family of PI3K-related protein kinases (PIKKs) includes mammalian Target of Rapamycin (mTOR), a serine/threonine kinase able to capture and transduce signals from different stimuli, such as the presence of amino acids, energy, oxygen, growth factors, and stress [33,34]. In mammals, there are two mTOR complexes with different localization and functions: Complex 1 (mTORC1) and complex 2 (mTORC2). mTORC1, which can be directly activated by the PI3K pathway or by PI3K-independent mechanisms, includes mTOR and the scaffolding protein RAPTOR (regulatory-associated protein of mTOR). mTORC1 phosphorylates eukaryotic translation initiation factor 4E-binding protein (4E-BP) and p70 ribosomal S6 kinase (S6K), leading to a modulation of ribosome biogenesis and protein synthesis, thus promoting cell growth and division [35,36,37]. mTORC2, containing the scaffolding protein RICTOR (rapamycin-insensitive companion of mTOR), is activated by growth factors and PI3K signaling, through still unknown processes. mTORC2 activity guides cytoskeleton organization, cell survival, and lipid metabolism, phosphorylating Akt kinase [38].

Abnormal activation of the PI3K/Akt/mTOR pathway has been associated with the development of several types of cancers, including GBM [22,29]. The PI3K/Akt/mTOR signaling is indeed activated in most GBM patients [3,39]. This pivotal pathway controls multiple downstream effectors engaged in a plethora of pro-tumorigenic effects, such as cellular survival, proliferation, protein translation, and motility. The PI3K pathway is affected by alterations of several signaling proteins, such as phosphatase and tensin homolog (PTEN) loss of function and EGFR amplification/mutation, two hallmarks of GBM pathogenesis [40]. Notably, no *Akt* and *mTOR* activating mutations have been observed in GBM, suggesting that changes in the functionality of these kinases are linked to the loss of function of PTEN and/or to alteration of RTK signaling [41]. *PTEN* acts as a tumor suppressor gene that encodes for a protein phosphatase, which negatively regulates intracellular levels of PIP3, and subsequently, PI3K/Akt pathway. Therefore, loss of *PTEN* function leads to an increase of Akt activity, which in turn triggers mTOR activity, thus stimulating cell proliferation and survival [42,43,44]. RTKs trigger class IA PI3Ks either directly or by interaction with the insulin receptor substrate (IRS) proteins, a family of adaptor molecules involved in the extracellular signals transduction from receptors to downstream proteins [45]. This interaction results in the production of PIP3, allowing several PIP3-dependent serine-threonine kinases activation, such as PI-dependent protein kinases 1 and 2 (PDK1 and PDK2), which in turn activate Akt, connecting growth factors signaling with cell growth, proliferation, metabolism, and survival [32,46]. It has been demonstrated that EGFR is often continuously activated in GBM. In addition, its internalization is impaired, as well as its recycling to the cell surface: These mechanisms contribute to PI3K hyperactivation [47]. In conclusion, RTK/PI3K/Akt signaling pathway is permanently activated in glioma cells, thus promoting cancer development [42].

## 4. RTK/PI3K/mTOR Axis and TMZ-Resistance

Even with the addition of new therapies, the alkylating agent Temozolomide (TMZ) remains the mainstay of treatment for HGG. Unfortunately, over 50% of GBM patients treated do not respond to the therapy, likely due to the highly heterogeneous and mutation-prone nature of GBM (oppure glioblastoma vs. temozolomide: Can the red queen race be won?). It is also quite common for these tumors to develop resistance to TMZ, which can either be an inherent characteristic or acquired after initial treatment [48]. As TMZ resistance is a clinically meaningful obstacle that must be overcome for the successful treatment of GBM, an in-depth understanding of the molecular mechanisms (and of the cellular populations) that drive resistance is needed. In addition to the activity of DNA repair enzymes, the dysregulation of specific molecular pathways contributes to TMZ resistance [48]. Among these altered pathways, alterations of RTKs, including their genetic mutations or an abnormal expression, have been involved in drug resistance in CNS tumors. As an example, EGFR amplification and the EGFRvIII mutated form have been implicated in many resistance mechanisms. RTKs, in fact, signal through two major downstream pathways, PI3K/Akt and MAPK/Erk, both altered in the majority of GBM as stated before [3]. Several downstream targets of Akt have been found to be implicated in specific mechanisms of TMZ resistance, including pyruvate dehydrogenase kinase 1 (PDK1), hypoxia-inducible factor 1 (HIF-1), forkhead O3 (FoxO3a), NF-kB, and other apoptotic regulators [49].

It is known that GBM, similarly to other solid tumors, preferentially utilizes glycolytic pathways for metabolism over oxidative phosphorylation, a shift referred to as the Warburg effect. PDK1 is thought the gatekeeper of glucose oxidation as it inactivates pyruvate dehydrogenase (PDH), inhibiting the metabolism of pyruvate in the Krebs cycle. An increased PDK1 expression is thought to be a hallmark for TMZ-resistance through supporting PDH inactivation in GBM cells [50].

The transcription factor HIF-1, which can be activated by PI3K/Akt, modulates the expression of several glycolytic genes and plays a key regulatory role in apoptosis, thus promoting chemoresistance [49]. It is known that low oxygen levels in the tumor stabilize transcription factor HIF-1, which translocates to the nucleus where it activates the vascular endothelial growth factor (VEGF) gene transcription, thereby increasing angiogenesis. The increased level of VEGF and VEGF receptor in GBM tumors gives rise to the highly vascularized nature of GBM.

Another important target of Akt implicated in drug-resistance is the transcription factor NF-κB, which has been identified as an oncogene in glioma, and its overactivation is expectedly associated with poor prognosis. Upon RTK/PI3K/Akt pathway activation, NF-κB translocates to the nucleus, where it promotes transcription of pro-survival genes. Some studies hypothesize that DNA damage caused by TMZ activates ataxia telangiectasia mutated (ATM) kinase, which concurrently triggers DNA repair and inappropriate activation of NF-κB [51].

Another PI3K/Akt-mediated apoptotic target is the Bcl-2 family of proteins. The expression level of antiapoptotic proteins, as well as pro-apoptotic proteins, can determine the fate of cancer cells, and in turn, the susceptibility to chemotherapy. In GBM stem cells, overexpression of antiapoptotic Bcl-2/bak and inhibition of the pro-apoptotic protein Bad plays a role in the escape from chemotherapy-induced cell death. Furthermore, survivin, another apoptosis inhibitor has been shown to block the effect of TMZ-induced apoptosis, conferring TMZ resistance, and its specific inhibition has been shown to increase TMZ sensitivity [52].

Lastly, inhibition of the PI3K/Akt/mTOR pathway has been shown to promote autophagy, a self-degradative process by which cellular constituents are removed and targeted for degradation. The role of autophagy in cancer onset and progression has been largely controversial as it may lead to cancer death or cancer survival depending on the tumoral type and stage. In GBMs, autophagy induced by TMZ is thought of as a survival and protective mechanism, and is generally considered as a mechanism of chemoresistance [53]. However, we recently demonstrated that mTOR inhibition, and in turn, autophagy induction potentiate the antiproliferative effect of TMZ in GBM models [54]. Therefore, further studies are necessary to dissect the role played by mTOR and autophagy on TMZ response.

By summarizing, it is likely that the alterations of one or some of the many downstream targets of the RTK/PI3K/mTOR pathway lead to an unbalance of the cellular processes that they regulate and creates a cellular environment that predisposes to drug-resistance. All these key molecular players should be kept in mind when designing targeted treatments or molecular profiling of GBM tumors to avoid the development of resistance to TMZ.

## 5. Targeting the RTK-PI3K-Akt-mTOR Axis

The identification of the main molecular mechanisms involved in oncogenesis represents the first step for the development of therapies that can be used in the treatment of cancer. Given that GBM is characterized by abnormal EGFR/PI3K/mTOR signaling activation in most cases, this pathway gained great interest in biomedical research, founding the rationale for novel therapeutic approaches. Several compounds have been identified so far, with the capability of targeting single or different components of the pathway. Notably, a number of clinical trials tested the safety profile and the efficacy of these drugs, both as single agents or in combination with standard therapeutic approaches, such as TMZ administration and radiotherapy [45,55].

### 5.1. RTK Inhibitors

RTK-targeting therapeutic strategies have been introduced in cancer clinical practice in 2001, when the Food and Drug Administration (FDA) approved Imatinib as a first-line agent to treat chronic myeloid leukemia (CML). Subsequently, RTK-targeted approaches have also been tested for efficacy in patients with glioma (Table 1); however, they achieve only a moderate antitumor activity so far [21,56]. EGFR, VEGFR, and PDGFR family members represent the major druggable targets, and a variety of RTKs inhibitors (TKIs) have been developed and employed in newly diagnosed or recurrent GBM [56].

#### 5.1.1. EGFR-Directed Therapies

EGFR mutations result in activation of the downstream signaling pathways PI3K/Akt and RAS/Erk in GBM cells, regardless of the binding to EGF. Starting with this notion, EGFR has been considered a very interesting target to treat diverse cancer types, including GBM (Table 1) [57,58,59].

The first-generation of EGFR inhibitors, Gefitinib and Erlotinib, as well as the second-generation EGFR inhibitor Afatinib, showed the ability to efficiently inhibit GBM cell growth, division, and angiogenesis in in vitro studies [60]. Gefitinib and Erlotinib are reversible and selective small-molecule tyrosine kinase inhibitors, which competitively block the binding of ATP to the EGFR tyrosine kinase domain, thus preventing the autophosphorylation and the subsequent downstream signaling pathway [61].

Afatinib, an oral bioavailable anilinoquinazoline compound, irreversibly inhibits EGFR and HER2 by its covalent binding to Cys 773 and Cys 805 residues, respectively [62]. Despite the promising in vitro results, including inhibition of GBM cell proliferation, growth, and angiogenesis, EGFR inhibitors have not exhibited therapeutic effectiveness in clinical trials. Gefitinib did not prolong survival in a phase II trial of recurrent GBM and in phase I/II trials of recently diagnosed GBM combined with radiotherapy [63]. Erlotinib did not show therapeutic efficacy and caused severe side effects in GBM patients [64,65]. Furthermore, the second-generation EGFR inhibitor Afatinib was not effective in inducing any significant clinical outcome in the treatment of primary and recurrent GBM [66].

The lack of clinical efficacy may reside in the fact that high concentrations of these inhibitors are required to inhibit the proliferation of GBM cells in vitro. Furthermore, these compounds are not able to efficiently cross the blood-brain barrier (BBB). Hence, BBB penetration is one of the main concerns that should be addressed to design successful pharmacological approaches against brain tumors.

The third-generation EGFR inhibitor Osimertinib (AZD9291) was initially approved to treat lung cancer with good therapeutic effects [67]. In addition, its capability to cross the BBB confers therapeutic effectiveness also in brain tumors [68].

P-glycoprotein (P-gp) and breast cancer resistance protein (BCRP) transporters contribute to blocking the passage of various substances across the BBB [69]. In contrast to other EGFR-TKIs, in fact, AZD9291 then quickly penetrates the brain, despite it is a P-gp and BCRP substrate [70]. Studies conducted on an animal model demonstrated that AZD9291 penetrates through the BBB and that its concentration in brain tissue is 5–25 times higher than in plasma [71]. Compared to other EGFR inhibitors, AZD9291 exhibited a good capability to inhibit cancer cell growth in a mouse model with brain metastases of lung cancer [70]. A recent study evaluated the effects of AZD9291 on GBM cell proliferation, migration, invasion, as well as its therapeutic efficacy in an intracranial mouse model of GBM. The results demonstrated that AZD9291 inhibited GBM cell proliferation to a greater extent than the other EGFR-TKIs and prolonged the survival of GBM-bearing mice [72]. These effects probably occur through a sustained blockade of the EGFR/ERK pathway. In addition, the authors suggest that AZD9291 may represent a good candidate in the treatment of GBM, both alone or in combination with other drugs, given its good BBB permeability, safety profile, and tolerance [72].

Therapies based on biological drugs (i.e., antibodies) were shown to be powerful tools in the treatment of different types of cancers. Conversely, their efficacy in GBM is very scarce or even absent [73,74,75]. To date, several antibodies have been produced to target wild-type EGFR and EGFRvIII, including Cetuximab, Panitumumab, and Nimotuzumab [76,77]. It has been observed that Cetuximab treatment significantly reduced cell survival and proliferation in EGFR-mutated GBM [78]. On the other hand, in a phase II study, patients were divided according to their EGFR amplification status, and both groups received intravenous Cetuximab, but the effect was limited to a median survival of 5 months. This discrepancy suggested that EGFR status is not predictive for survival [79]. In a phase III study, patients underwent Nimotuzumab treatment combined with radiotherapy. Despite there was not a significant difference in patients’ survival, EGFR amplification and unmethylated O6-alkylguanine DNA alkyltransferase (MGMT) were associated with higher median survival [77].

#### 5.1.2. PDGFR-Directed Therapies

Enhanced PDGFR activity caused by genetic mutations characterizes the refractoriness to therapies of a proneural subgroup of gliomas. Therefore, PDGFR inhibitors have been developed to treat this type of glioma (NCT01140568). However, although the data from both in vitro and animal experiments support the potent inhibitory effects of PDGFRi on GBM cells, clinical trials of single PDGFRi have failed to show encouraging antitumor effects, which might result from the rapid emergence of resistance to PDGFRi [80,81]. Imatinib, a tyrosine kinase inhibitor of PDGFR, c-KIT, and BCR-ABL, improved sensitivity to chemotherapy in vitro, but without showing a meaningful antitumor activity in high-grade gliomas in clinical trials [60,82]. Coherently, phase II studies evaluated a therapeutic regimen based on a combination of Imatinib and hydrossyurea, showing irrelevant antitumor activity in patients with either recurrent/progressive low-grade glioma or recurrent GBM [82].

Dasatinib, an ATP-competitive inhibitor of PDGFR, c-KIT, BCR-ABL, and SRC, has been evaluated as a monotherapy in patients with GBM relapse for a phase II study (NCT00423735) and resulted ineffective in recurrent GBM. Moreover, in a phase I/II study, Dasatinib plus Lomustine (CCNU) in patients with GBM relapses displayed no considerable effects [83]. A second-generation PDGFR inhibitor Tandutinib has been assessed in a phase I/II trial (NCT00379080). Importantly, side effects on neuromuscular junction have been observed that could limit the use of PDGFR inhibitors [84].

#### 5.1.3. VEGFR-Directed Therapies

VEGFR family includes VEGFR1, VEGFR2, and VEGFR3 that are potent angiogenic inducers. For this reason, several VEGFR inhibitors have been developed and applied in cancer therapy. Preclinical and clinical studies on GBM have also been conducted.

Cediranib (AZD2171) is a pan-VEGFR inhibitor that also inhibits the activity of other RTKs, such as c-Kit and PDGFRA/B. In Phase II trials, Cediranib treatment induces tumor vessel normalization and edema reduction, which is correlated to improved progression-free survival (PFS) in newly diagnosed GBM patients [85,86]. Other VEGFR inhibitors, including Aflibercept, BIBF 1120, Pazopanib, AMG 386 (Trebananib), and Vandetanib, have been tested in combination with other drugs in phase I/II trials [87]. Among these, Aflibercept, which acts as a decoy receptor for VEGF ligands, has shown limited success in phase II trials for recurrent GBM patients [88]. On the contrary, the mAb against VEGF, Bevacizumab (Avastin^®^), is currently used in patients with GBM, usually in combination with other drugs, resulting in objective radiographic responses and improvement in PFS [89]. Since its approval by FDA in 2004, over 60 countries have employed Bevacizumab in the treatment of the progressive disease [90,91,92]. Notably, two Phase III trials indicated an improved PFS, but not OS, of newly diagnosed GBM patients when Bevacizumab was used in combination with the standard protocol (TMZ and RT) [93,94].

#### 5.1.4. Therapies Directed against Other RTKs

IGF-1R is considered another interesting target for GBM treatment. In the last years, a number of small-molecule inhibitors against IGF-1R have been tested on GBM cells in vitro and in vivo. Among them, PQ401, BMS-536924, or PPP (picropodophyllin/AXL1717) can suppress the growth and migration of glioma cells, while GSK1838705A or NVP-AEW541 induced apoptosis either alone or in association with other chemotherapeutic drugs [95,96,97,98,99]. Other inhibitors, such as OSI-906 and BMS-754807, also showed good efficacy on GBM cells in vitro [100].

Although FGFR mutations are rare in GBM, studies suggest that modifying FGFR signaling influences GBM progression and patient survival [101]. Currently, FGFR tyrosine kinase inhibitors are under active investigation, exploring the therapeutic potential of this signaling pathway [102]. Small-molecule inhibitors, such as Lenvatinib, Ponatinib, Dovitinib, and Brivanib, are non-specific compounds and also target other RTKs, while PD173074, BGJ398, AZ4547, and JNJ-493 are highly selective for FGFR [103]. In a recent in vitro study, a large-scale shRNA screen was used to identify FGFR signaling as a target in pediatric glioma, proving that the reduction of glioma growth provided by these molecules is better than TMZ [104].

In December 2019, a clinical trial aimed at assessing the putative effectiveness of BGJ398 in patients with recurrent GBM was completed, but so far, no results have been published [105]. An ongoing phase I/II trial involving TAS-120 is currently recruiting patients with advanced solid tumors, with and without FGF/FGFR-related abnormalities (NCT01975701).

### 5.2. mTOR Inhibitors

Since mTOR plays important roles in regulating cancer cell growth, metabolism, and protein synthesis, the inhibition of this complex is regarded as another interesting pharmacological approach to reduce the effects of a constitutive PI3K/Akt activation in GBM (Table 2). The main mTORC1 inhibitors include Rapamycin (Sirolimus) and its analogs, such as RAD001 (Everolimus), CCL-779 (Temsirolimus), and AP23573 (Ridaforolimus) [106]. Rapamycin inactivates mTORC1 by altering its kinase conformation. Despite Rapamycin and its analogs demonstrate effectiveness per se in both in vitro and in vivo models [107,108], mTORC1 inhibition is largely compensated by hyperactivation of Akt and mTORC2 activity [109]. Coherently with this notion, despite Rapamycin exhibits antitumor activity in a phase I trial for patients with recurrent GBM and PTEN loss (NCT00047073) [110], phase II clinical trials for Rapamycin analogs did not achieve the same positive results (NCT00515086, NCT00016328, NCT00022724, and NCT00087451) [111,112,113,114,115]. Furthermore, limited effectiveness could also result from feedback loops and from the crosstalk with other signaling pathways. This is probably why the combination of Rapamycin analogs with other drugs has been given greater prominence [115].

mTORC2 blockade may be more advantageous than mTORC1 inhibition, as it could directly suppress Akt phosphorylation, although without disrupting the mTORC1-dependent feedback loops [116,117]. AZD8055, an ATP-competitive inhibitor, reduced S6 and Akt phosphorylation in vivo, resulting in a reduction of cancer growth [118]. Thus, AZD8055 could represent a more auspicious therapeutic strategy than Rapamycin and its analogs [119].

Differently, a recent study showed that mTOR suppression, mediated by small-molecule compounds capable of inhibiting both mTORC1 and mTORC2, can present both advantages and disadvantages at the same time. In fact, the inhibitors of mTORC1/2 like Torin2, NVP-BEZ235, and INK-128 are capable of contextually block the activity of mTORC1 and mTORC2 complexes, and this inhibition leads to an enhanced arrest of cell growth if compared to Rapamycin. However, these inhibitors do not cause cytotoxicity and appear to improve the survival of glioma cells in hypoxic and nutrient-deficient conditions. This protection from cell death appears to be mediated by the metabolic changes induced by mTOR inhibition, which may explain the resistance mechanisms recently identified [120].

Among the ATP-competitive mTORC1/2 inhibitors, Torin1 is a compound capable of hindering growth, motility, invasion, and survival of colon cancer stem cells in vitro, and of suppressing tumor growth in vivo and reducing angiogenesis, without affecting the survival of normal colon stem cells, suggesting its selectivity towards cancer cells [121].

**Figure 2:**
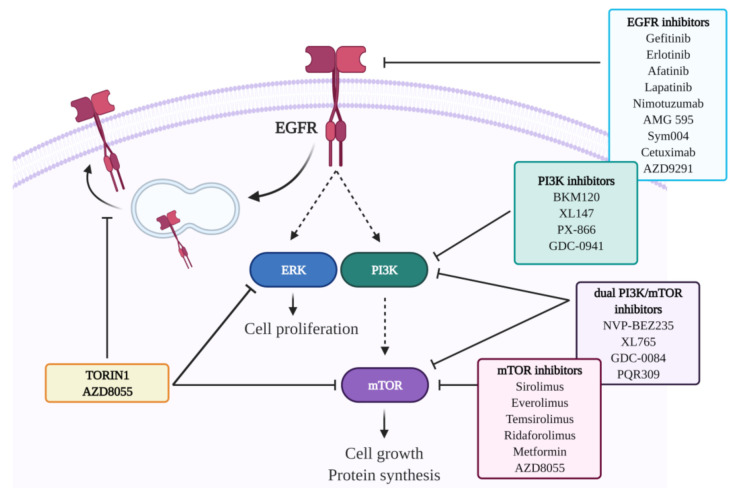
EGFR/PI3K/mTOR signaling pathway and inhibitory molecules used in preclinical and clinical studies for glioma therapy. Upon binding to its ligand or genetic alterations, EGFR is activated and triggers a series of cascade reactions. Among these reactions, the phosphorylation and the consequent activation of the ERK pathway and the PI3K/mTOR axis regulate cell growth, proliferation, motility, survival, transcription, and protein synthesis. Many molecules have been developed and employed targeting EGFR, PI3K, and mTOR proteins. Recently, mTOR inhibition by Torin1 and AZD8055 has been shown to mediate EGFR internalization and its delivery to lysosomes in GBM cells [54]. This image was created with BioRender software.

In this regard, we have recently shown that Torin1 can inhibit cell proliferation and sensitize GBM cells to TMZ in in vitro experiments [54]. The antiproliferative effect of Torin1 is likely mediated by ERK1/2 downregulation, which could be the consequence of the observed impairment of EGFR recycling to the cell surface [54] (Figure 2). In previous studies, we also demonstrated that both Torin1 and Rapamycin can impair cell migration and invasion, by reverting the EMT process and by impinging the Wnt/β-catenin pathway in GBM cells [122,123].

Recently, Torin2, which has better pharmacokinetic properties and an improved synthetic route than Torin1, has been synthetized, thus paving the way for new mTOR pharmacological inhibition [124].

### 5.3. PI3K Inhibitors

Genetic aberrations in GBM, including EGFR and PTEN alterations, leading to a dysfunction of the PI3K/Akt/mTOR pathway with effects on cell proliferation, metabolism, apoptosis, motility, and angiogenesis in GBM. RTKs or G protein-coupled receptors (GPCRs) activate the PI3K/Akt pathway. Particularly, class IA and IB PI3Ks essentially respond to the activation of RTKs and GPCRs, respectively [125]. Abnormal activity of the PI3K/Akt pathway promotes growth, tumor progression, and resistance to various drugs in GBM cells. Therefore, inhibition of PI3K in monotherapy or in combination with other drugs represents another card to play in the treatment of GBM.

PI3K inhibitors are usually classified into pan-PI3K, isoform-specific, and dual PI3K/mTOR inhibitors.

#### 5.3.1. Pan-PI3K Inhibitors

Pan-PI3K inhibitors include a list of molecules directed against all members of the PI3K family. One of the first generation pan-PI3K inhibitors is Wortmannin, characterized by little clinical use because of its insolubility, short half-life, and high toxicity in animal studies. New generation pan-PI3K inhibitors with greater safety have been developed and have been investigated in clinical trials (Table 3). Among these, Buparlisib (BKM120) is an oral bioavailable pan-PI3K inhibitor, which induces G2/M cell cycle arrest and apoptosis in GBM cells via microtubule misalignment and mitotic disruption in a p53-dependent manner [126]. It also facilitates tumor necrosis factor-related apoptosis-inducing ligand (TRAIL)- and Bcl-2 inhibitor-induced apoptosis in GBM cells through Noxa expression, sequestration of Mcl-1 by Noxa, and delivery of pro-apoptotic protein Bim and Bak from Mcl-1 [127,128]. Furthermore, preclinical studies show that BKM120 prevents the growth of intracerebral U87 MG GBM cell xenograft and extends the survival of tumor-bearing animals, with no apparent adverse effects [126,129]. Considering that BKM120 is well-tolerated (only mild treatment-related toxicity was observed) and permeable to the BBB, it is the most frequently used PI3K inhibitor in the clinical trials for GBM treatment. Patients with PTEN loss and/or PIK3CA mutations were not responsive to BKM120 treatment, thus BKM120 is also used in combination with other drugs or radiotherapy, and the safety, dose, and antitumor activity of these combinations are currently evaluated [125]. Another orally bioavailable pan-class IA PI3K inhibitor is Pilaralisib (XL147, SAR245408) [130]. XL147 showed dose-dependent antiproliferative effects on both GBM and breast cancer cells via PI3K and Akt inhibition in vitro. Presently, XL147 has entered phase I/II clinical trials in different types of cancers, such as breast, endometrial, lymphoma, and GBM [131,132,133,134,135]. In a phase I exploratory pharmacodynamic study, XL147 has been assessed in combination with XL765 (Voxtalisib, a dual PI3K/mTOR inhibitor) in patients with GBM relapses before surgical resection. These two drugs reduce S6 K1 phosphorylation and Ki67 expression, indicating that they have an appreciable BBB penetration ability and a potential effect on blocking GBM growth [135].

Sonolisib (PX-866) is a Wortmannin analog, pan-PI3K inhibitor, with a more potential antineoplastic activity than Wortmannin [136,137]. PX-866, used alone or in combination with other drugs, demonstrates pro-autophagic, anti-invasive, and antiangiogenic effects in GBM cells, likewise antitumor effects in intracranial GBM xenograft mice [138,139]. PX-866 suppressed cyclin D1 expression and activated Retinoblastoma protein Rb1, reducing GBM cell number in the G1 phase; it also induced autophagy favoring the conversion of LC3-I to LC3-II. The activity of this molecule also resulted in a decreased secretion of VEGF and in a reduced invasion rate in different cell lines. Importantly, PX-866 significantly slowed down tumor growth in a mouse model of intracranial U87 MG GBM xenograft, also improving the overall survival [138]. In addition, PX-866, in combination with the dinuclear platinum compound BBR3610, showed synergistic effects on GBM cell migration and lifespan of GBM-bearing mice [139]. Considering its excellent oral bioavailability, PX-866 entered clinical trials for many types of cancer, including ovarian, colorectal, prostate, head and neck cancers, melanoma, non-small cell lung cancer, and GBM [140].

Besides the above-mentioned compounds, different novel pan-PI3K inhibitors have been developed to date, and some of them may represent interesting options for GBM treatment. Chiefly, these molecules may suppress the proliferation of GBM cell lines, as well as subcutaneous U87 MG xenograft growth in animal models [141,142,143].

#### 5.3.2. Isoform-Specific PI3K Inhibitors

Specific inhibitors against p110 isoforms could possess less off-target effects and toxicity, thereby representing promising alternatives for GBM treatment. Several in vitro studies using traditional isoform-specific PI3K inhibitors display that class IA PI3K isoforms play different roles in glioma progression. Inhibition of p110α by the molecules PIK-75 or A66 is adequate to suppress GBM cell viability, migration, and invasion; inhibition of p110β by TGX-221 just blocks cell migration, and inhibition of p110δ by IC87114 or by CAL-101 mildly prevents cell proliferation and migration [144,145]. Because of the prominent role of p110α in RTK-mediated Akt signaling, inhibition of p110α could be an efficient approach to hinder GBM. Considering that both *PIK3CA* mutations and *PTEN* loss/mutation are commonly found in GBM, isoform-specific PI3K inhibitors, especially against p110α and p110β, could have prospective roles in the treatment of GBM characterized by these genetic mutations.

Despite different isoform-selective PI3K inhibitors, such as BYL719, MLN1117, CAL-101, GSK2636771, and CH5132799, entered phase I/II clinical trials to study their potential on solid tumors and hematologic malignancies, no clinical studies have yet been addressed on GBM patients [125].

#### 5.3.3. Dual PI3K/mTOR Inhibitors

The crosstalk and feedback regulation between PI3K and mTOR greatly restrict the therapeutic impact of PI3K or mTOR inhibitors. Thereby, dual PI3K/mTOR inhibitors are developed and are actually evaluated in clinical trials [125] (Table 3). These inhibitors include the molecules Dactolisib (NVP-BEZ235), Voxtalisib (XL765), GDC-0084 (RG7666), and PQR309.

NVP-BEZ235 is an oral bioavailable, reversing, ATP-competitive dual PI3K and mTORC1/2 inhibitor, largely used in preclinical studies on several cancers comprising GBM, breast, colorectal, and lung cancers [146,147,148,149]. It sensitizes GBM cells to radiotherapy and TMZ both in vitro and in vivo, by reducing the activation of Akt, increasing the expression of pro-apoptotic proteins Bax and Caspase-3, and blocking radiation-induced DNA damage repair [146,150]. Notably, the inhibiting effect on Akt activation is reversible, and an increased radiosensitization is just noted when U87 MG is treated with NVP-BEZ235 [45]. Moreover, NVP-BEZ235 promotes cell differentiation in neuronal and glial lines and abolishes the tumorigenicity of GBM stem-like cells [41,151]. These discoveries suggest that NVP-BEZ235, in combination with TMZ, radiotherapy, or other inhibitors, could represent a potential strategy for GBM treatment. NVP-BEZ235 has already entered phase I/II clinical trials in different cancers, such as breast cancer, prostate cancer, pancreatic neuroendocrine tumor, and leukemia [152,153,154]. In a phase I study on patients with leftover solid tumors, NVP-BEZ235 was commonly well tolerated with slight dose-limiting toxicity [154]. A phase IB clinical trial on patients with leftover solid tumors, comprising GBM, highlighted that NVP-BEZ235 plus Everolimus showed limited effectiveness and unacceptable adverse events, such as fatigue, diarrhea, nausea, mucositis, and increase of liver enzymes in serum [154]. Recently, NVP-BEZ235 has been included in a phase IIB study (NCT02430363), in combination with Pembrolizumab (MK-3475, a PD-1 monoclonal antibody) to treat GBM patients. The rationale of this combined therapy resides in the fact that, after PD-1 inhibition by MK-3475 administration, the PI3K/Akt pathway is reduced, and the activation of T cells repressed, thus promoting the tumor immune escape [125].

Voxtalisib (XL765, SAR245409) is also a powerful ATP-competitive, oral bioavailable, BBB-permeable PI3K/mTOR inhibitor with high affinity for p110γ, and an additional inhibitory activity against DNA protein kinase [155]. It shows a solid antiproliferative activity in a group of GBM cell lineages through reduction of Akt, GSK3β, and p70S6 K phosphorylation, decreased cyclin D expression, and induction of cell cycle arrest in the G1 phase. In relation to different genetic modifications characterizing tumor cells, XL765 may have diverse effects on cellular proliferation. Cell lineages with PIK3CA mutations or amplifications, but wild-type RAS, are more susceptible to XL765; conversely, cells harboring RAS mutations are relatively unresponsive to XL765 although in the presence of PIK3CA mutations. Moreover, XL765, when administered as a single agent or in combination with TMZ, distinctly nullifies the growth of subcutaneous or intracranial GBM xenografts, and extends survival of tumor-bearing mice, underlying its powerful anti-GBM activity and BBB penetration ability [155,156]. In a phase I study aimed at assessing dose effects and safety profiles, XL765-induced toxicity was acceptable in patients with solid tumors [157]. A phase I dose increase study performed on patients with HGGs, the effects of XL765 in combination with TMZ, with or without simultaneous radiotherapy, were assessed. The different treatment regimens exhibited similar negative effects plus thrombocytopenia [158]. In a different phase I clinical trial, the ability of XL765 to cross the BBB has been proven in patients with GBM relapse. In the same study, an efficient reduction of phosphorylation of the mTOR substrate S6K was observed, as well as a reduced expression of the proliferation marker Ki67 [135].

GDC-0084 (RG7666) is a new PI3K/mTOR inhibitor able to cross the BBB. In vitro, it significantly prevents GBM cell proliferation. These results are in line with the fact that, in tumor-bearing mice, this compound efficiently blocks the U87 MG GBM growth by reducing Akt phosphorylation [159]. In addition, GDC-0084 presents a high brain-to-plasma ratio and tumor-to-plasma ratio, indicating its uniform distribution in the whole brain (NCT01547546) [160].

PQR309 is an ATP-competitive, BBB-permeable dual PI3K/mTOR inhibitor with powerful inhibitory activity on Akt and ribosomal protein S6 phosphorylation. In a non-randomized phase II study, the efficacy, safety, pharmacokinetic and pharmacodinamic effects of PQR309 are assessed in patients with progressive GBM (NCT02850744) [125].

Other new dual PI3K/mTOR inhibitors, such as NVP-BGT226, GSK2126458, GSK1059615, GDC-0980, VS-5584, PF-04691502, and PKI-587, entered phase I/II clinical trials for leftover solid tumors. Nevertheless, no clinical studies have yet been performed in GBM patients [125].

## 6. Limitations of RTK/PI3K/mTOR-Targeting Therapies

### 6.1. Resistance to RTKs

The mechanisms of resistance at the root of RTK-targeted therapy depend on (1) intratumoral heterogenicity and cooperation of various RTKs and their downstream signaling pathways; (2) intertumoral heterogenicity of RTK expression and activity within TME; (3) the treatment-induced adaption of TME, including secondary hypoxia, accumulation of GSC and immune suppression [24].

The experience with RTKs-based therapies has revealed that, even if receptors are efficiently de-activated by inhibitors, the treatment may result clinically ineffective because of the activation of regulatory circuits, promoting alternative activation of downstream pro-tumoral signaling [24]. This phenomenon occurs in EGFR- and in VEGFR-based therapies: In particular, upon treatment with VEGFR inhibitors, other angiogenic factors, such as FGF and PDGF, activate converged signaling cascades involved in neovascularization [93,161]. In addition to alternative activation of downstream pathways, cooperation between RTKs may compensate for the loss of the specifically targeted receptor [20]. For example, EGFR inhibitors attenuate the transcriptional inactivation of PDGFR operated by EGFRvIII in tumor cells, thus enabling cells to switch to PDGFR-mediated pro-tumoral signaling [21]. Furthermore, gene expression profiles identify that anti-VEGF therapy may induce c-Met activation, which in turn represents a possible mediator of drug resistance [162].

It is conceivable that the link between RTKs and TME can contribute to resistance of RTKs-based or combinational therapies [56,163,164]. For instance, the treatment with the VEGFR2 inhibitor Valatanib, which initially induces transient benefits as an antiangiogenic drug, mediates hypoxia and increased activation of pro-angiogenic cytokine/chemokine pathways involved in the promotion of tumor progression [165].

Resistance to single or combinatorial treatments based on RTKs is also associated with the accumulation of GSCs and with immune suppression. GSCs cells can differentiate into endothelial cells and contribute to the formation of neovascular structures required to provide nutritional support to hypoxic tumor areas [166,167].

In order to overcome the onset of RTKs resistance, novel strategies based on the combination of multiple inhibitors blocking different targets of the same pathway (vertical inhibition) or the blockade of key proteins involved in diverse signaling pathways (horizontal inhibition) have been established [168]. As described above, these strategies may include the co-administration of multiple drugs or the employment of single agents with multi-target ligand properties [169,170].

Noteworthy, most clinical studies on RTKs targeted therapy lack sufficient information regarding the measurement of intratumoral drug concentration, the target engagement, and the real-time degree of RTK inhibition. The future design of combination therapies should consider such information, in addition to monitoring the dynamic tumor profiles [24].

### 6.2. Limitations of the mTOR-Inhibiting Approaches

Akt and mTOR activation have been associated with a poor prognosis in GBM [171]. Following mTORC1 inhibition, a compensatory Akt activation by unregulated mTORC2 occurs: It is believed that this may be one of the reasons for the inefficacy of mTORC1 inhibitors. The limited capability to cross the BBB is another Achille’s heel of these compounds mTOR inhibitors. Indeed, although preclinical studies had furnished a strong rationale for developing mTOR-based strategies, a randomized phase II clinical trial showed that Everolimus, a selective inhibitor of mTORC1, was not only ineffective in GBM, but also revealed unexpected toxicity and resulted in a reduced median OS of patients [172]. Some differences between control and experimental arms of the trial may account for the significant difference in the OS observed in the two groups, and a different trial design may reach different outcomes [10]. Differently from mTORC1-directed inhibitors, mTORC1/mTORC2 dual inhibitors do not elicit the feedback activation of the PI3K/Akt signaling. Ongoing clinical trials with dual inhibitors should clarify the critical role of mTOR in GBM treatment.

Although the results obtained so far are not encouraging, the PI3K/Akt/mTOR axis still retains a great interest as a possible target for therapeutic intervention in GBM, and novel compounds are continuously developed and tested to overcome the limitations observed with the Rapamycin derivatives (rapalogs).

Next-generation ATP-competitive mTOR inhibitors with an affinity for both mTOR complexes have been developed, but data exploring their effects on GBM metabolism are still scarce. In comparison with Rapamycin, dual mTORC1/2 inhibition led to stronger growth inhibition, which partly coincided with cell cycle arrest [120]. However, dual mTORC1/2 inhibitors did not cause cytotoxicity in cancer cells, and affect cellular metabolism, leading to an increased tolerance to nutrient deprivation and hypoxia [120]. These results underline the importance of the preclinical experimental settings that adequately reproduce the TME context to assess the potential of pharmacological inhibitors as a therapeutic option.

## 7. Combination Strategies within the EGFR-PI3K-mTOR Pathways to Improve Therapeutic Efficacy

A multitarget treatment may be a good solution when certain subclones of the tumor become resistant to single treatment also because of the occurrence of genetic mutations; therefore, an option to overcome resistance is to selectively act on these mutations. Targeting multiple components of the EGFR-PI3K-mTOR axis could be an efficient therapeutic approach in GBM and other tumors associated with these alterations. However, combination therapies blocking EGFR and downstream PI3K signaling in gliomas exhibited limited effectiveness [30] (Table 4).

Therapeutic effects derived from the combination of Erlotinib with Rapamycin or the combination of Erlotinib and Bevacizumab on GBM relapses were evaluated, but the results were disappointing [65]. The combination of Erlotinib with Sirolimus or Temsirolimus has also been tested in clinical trials (NCT00112736 and NCT0062243), but both of them did not show promising results [176,177]. On the other hand, a phase II clinical trial demonstrated that a combination therapeutic regimen based on Everolimus and Bevacizumab administration was effective as first-line therapy for GBM (NCT00805961) [65,176]. A phase I/II clinical trial in recurrent HGGs (NCT01051557) was characterized by a combination of the Akt inhibitor Perifosine and Temsirolimus, which together inhibited murine GBM growth regardless of the PTEN status [178,179].

Pictilisib (GDC-0941) is derived from the dual PI3K/mTOR inhibitor PI-103, but it inhibits class I PI3K with less effect on mTOR. It displays a similar antiproliferative activity to PI-103 against human cancer cells, comprising U87 MG GBM cells [180]. Furthermore, GDC-0941 elicits a marked and prolonged inhibition of Akt phosphorylation, accompanied by the suppression of tumor growth in subcutaneous U87 MG xenograft mice [180]. In combination with the Bcl-2 family inhibitor ATB-263, GDC-0941 exhibited synergistic effects in mediating loss of mitochondrial membrane potential, inducing GBM cell apoptosis and deleting sphere formation in GBM stem-like cells. These events are mediated by a decreased Akt phosphorylation and Mcl-1 expression [173]. In a preclinical study, the BBB penetration properties and the cerebral distribution of two other PI3K inhibitors (GDC-0941 and GNE-317) were examined in U87 MG and GS2 intracranial GBM xenograft mouse models. GNE-317 displayed superior BBB penetration in both models. Otherwise, GDC-0941 hardly crossed the intact BBB, suggesting that it could be difficult for this molecule to reach the distant part of GBM [174]. In addition, an in vivo study also revealed that the combination of GDC-0941, Irinotecan, Sunitinib, and TMZ does not significantly prolong the survival of mice with GBM xenograft, probably because of the poor BBB permeability of GDC-0941 [175]. Despite these negative results, an ongoing phase IIB clinical trial in patients with recurrent GBM is assessing the antitumor activity of Pembrolizumab (MK-3475, a PD-1 monoclonal antibody) alone or in combination with GDC-0941 or other PI3K inhibitors, including NVP-BEZ235 (NCT02430363).

## 8. Conclusions and Future Directions

Gliomas, particularly HGGs, are the most challenging malignancies, due to their infiltrative nature, tendency to recurrence, and poor response to treatments. All these features greatly depend on tumor heterogeneity, including inter- and intratumoral mutational pattern variation and intratumoral histological variations [21]. Surgical resection followed by adjuvant chemoradiotherapy is the gold standard treatment for this kind of tumors; however, its effectiveness is limited. Indeed, although advances in neurosurgical techniques and in radiation therapy are beneficial for patients, the modest effects of chemotherapy are still challenging. Moreover, tumor recurrence is associated with the onset of therapy resistance; it is therefore critical to identify effective and well-tolerated pharmacological approaches capable of inducing durable responses in the appropriate patient groups. The RTK/PI3K/Akt/mTOR signaling pathway is a crucial player in the genesis and progression of gliomas (Figure 2). Although preclinical studies showed promising results about the prospective effectiveness of RTK/PI3K/mTOR inhibitors, several clinical trials based on single or combination therapy regimens highlighted limited or no efficacy (Figure 2). The therapeutic failure of these studies can be explained by numerous reasons, such as the scarce BBB permeability of certain compounds, or the occurrence of resistance/tolerance mechanisms following the chronic administration of the drug. Furthermore, no systematic studies were aimed at addressing the tumor heterogeneity, the protective interaction with the microenvironment, as well as diverse posology adjustments [9,10,24]. Since RTK/PI3K/mTOR is one of the most pivotal pathways regulating cell growth and survival in cancer biology, its targeting still remains a strong rationale for developing strategies against gliomas. Intriguingly, we recently demonstrated a peculiar feedback loop between mTOR inhibition and RTKs trafficking/expression. Chiefly, the mTOR inhibitors Torin1 and AZD8055 can induce EGFR internalization and lysosomal delivery in GBM cells via an Src-dependent mechanism (Figure 2) [54]. EGFR delocalization is accompanied by ERK1/2 inactivation, which is likely responsible for a substantial reduction of cell proliferation and the potentiation of the antiproliferative effect of TMZ [54]. These results, together with previous evidence showing an mTOR-dependent impairment in invasion capabilities of GBM cells [122], suggest that the effects of mTOR inhibition can be further exploited to counteract glioma proliferation, migration, and invasion. Future rigorous clinical studies targeting one or more players of this signaling cascade should include molecular biomarkers, bioptic profile monitoring, and a well-adjusted drug delivery system that might ensure therapeutic efficacy and response prediction.

## Figures and Tables

**Table 1:** Ongoing clinical trials on adult gliomas targeting RTKs (data from http://clinicaltrials.gov, accessed on 30 April 2021).

RTK	Drug Name	Type of Glioma (Number of Patients Enrolled)	Adjuvant Therapy	Clinical Trial
EGFR	Gefitinib	Recurrent GBM (*N* = 22)	None	Phase II NCT00250887
		GBM (*N* = 158)	Radiotherapy	Phase I/II NCT00052208
		GBM (*N* = 36)	Radiotherapy	Phase II NCT00238797
		Gliomas and meningiomas (*N* = 105)	None	Phase II NCT00025675
	Erlotinib	GBM (*N* = 110)	None	Phase II NCT00337883
		GBM, gliosarcoma (*N* = 66)	TMZ, radiotherapy	Phase II NCT00187486
	Afatinib	Refractory solid tumors (*N* = 60)	None	Phase II NCT00875433
		GBM (*N* = 36)	TMZ, radiotherapy	Phase I NCT00977431
		GBM (*N* =151)	TMZ	Phase II NCT00727506
	Lapatinib	GBM (*N* = 24)	None	Phase I/II NCT00099060
		Malignant brain tumors (*N* = 9)	None	Phase II NCT00107003
	Nimotuzumab	GBM (*N* = 45)	None	Phase II NCT00561873
		GBM (*N* = 150)	TMZ, radiotherapy	Phase III NCT00753246
	AMG 595	GBM (*N* = 32)	None	Phase I NCT01475006
	Sym004	Malignant glioma (*N* = 43)	None	Phase II NCT02540161
	Cetuximab	GBM (*N* = 46)	TMZ, radiotherapy	Phase I/II NCT00311857
	Osimertinib (AZD9291)	Metastatic brain tumors (*N* = 112)	None	Phase II NCT02971501
PDGFR	Nilotinib	Glioma (*N* = 38)	None	Phase II NCT01140568
	Imatinib	Brain and CNS tumors (*N* = 112)	None	Phase II NCT00039364
		GBM, Gliosarcoma (*N* = 64)	Hydroxyurea	Phase II NCT00615927
	Dasatinib	GBM, gliosarcoma, reccurrent adult brain tumors (*N* = 64)	None	Phase II NCT00423735
		GBM (*N* = 28)	Lomustine (CCNU)	Phase I/II NCT00948389
	Tandutinib	GBM, gliosarcoma, primary and recurrent brain tumor (*N* = 60)	None	Phase I/II NCT00379080
VEGFR	Cediranib (AZD2171)	GBM and gliosarcoma (*N* = 46)	TMZ, radiotherapy	Phase I/II NCT00662506
	Aflibercept	Anaplastic astrocytoma, anaplastic oligodendroglioma, GBM, gliosarcoma, mixed glioma, recurrent brain tumors (*N* = 61)	TMZ, radiotherapy	Phase I NCT00650923
	BIBF 1120	Recurrent GBM (*N* = 25)	None	Phase II NCT01251484
	Pazopanib	GBM, gliosarcoma, recurrent brain tumor (*N* = 35)	None	Phase II NCT00459381
FGFR	TAS-120	Brain tumors (unavailable)	None	Phase I/II NCT02052778

**Table 2:** Ongoing clinical trials on adult gliomas targeting mTOR kinase (data from http://clinicaltrials.gov, accessed on 30 April 2021).

Drug Name	Type of Glioma (Patients Enrolled)	Adjuvant Therapy	Clinical Trial
Sirolimus	GBM (*N* = 13)	None	Phase I/II NCT00047073
Temsirolimus	Brain and CNS tumors (*N* = 12)	None	Phase I NCT00784914
	Brain and CNS tumors (*N* = 49)	None	Phase I/II NCT00022724
	GBM (*N* = 33)	None	Phase II NCT00016328
Ridaforolimus	Glioma (*N* = 11)	None	Phase I NCT00087451
Metformin	GBM (*N* = 144)	None/TMZ	Phase I NCT01430351
AZD8055	GBM (*N* = 22)	None	Phase I NCT01316809

**Table 3:** Ongoing clinical trials on adult gliomas targeting PI3K (data from http://clinicaltrials.gov, accessed on 30 April 2021).

Classification	Drug Name	Type of Glioma (Patients Enrolled)	Adjuvant Therapy	Clinical Trial
Pan-PI3K inhibitors	Buparlisib (BKM120)	GBM (*N* = 65)	None	Phase I/III NCT01339052
		GBM (*N* = 38)	TMZ, radiotherapy	Phase I NCT01473901
	Pilaralisib (XL147, SAR245408)	GBM (*N* = 40)	None	Phase I/II NCT01240460
	Sonolisib (PX-866)	GBM (*N* = 34)	None	Phase I/II NCT01259869
	Pictilisib (GDC-0941)	GBM (*N* = 58)	None	Phase I/II NCT02430363
Dual PI3K/mTOR inhibitors	Voxtalisib (XL765, SAR245409)	GBM (*N* = 40)	None	Phase I/II NCT01240460
		HGGs (*N* = 54)	TMZ	Phase I NCT00704080
	GDC-0084	HGGs (*N* = 29)	None	Phase I NCT01547546
	PQR309	GBM (*N* = 28) (*N* = 70) (*N* = 10)	None	Phase I/II NCT01940133 NCT02483858 NCT02850744

**Table 4:** Ongoing clinical trials on adult gliomas based on a combined approach targeting RTKs/mTOR/PI3K axis (data from http://clinicaltrials.gov, accessed on 30 April 2021).

Drug Name	Type of Glioma (Number of Patients Enrolled)	Combined Therapy	Clinical Trial
Erlotinib	GBM, gliosarcoma (*N* = 57)	+ Bevacizumab	Phase II NCT00671970
	Primary and recurrent brain tumors (astrocytoma, oligodendroglioma GBM, gliosarcoma, mixed glioma) (*N* = 69)	+ Temsirolimus	Phase I/II NCT00112736
Sirolimus	GBM(*N* = 32)	+ Erlotinib	Phase II NCT00672243
Sirolimus	Brain and CNS tumors (*N* = 19)	+ Erlotinib	Phase I NCT00509431
Temsirolimus	Recurrent HGGs (*N* = 36)	+ Perifosine	Phase I/II NCT01051557
Pictilisib (GDC-0941)	Recurrent GBM (*N* = 58)	+ Pembrolizumab (MK-3475)	Phase IIB NCT02430363
	GBM	+ ATB-263	Preclinical [173]
	GBM	+ GNE-317	Preclinical [174]
	GBM	+ Irinotecan, sunitinib and TMZ	Preclinical [175]
Dactolisib (NVP-BEZ235)	GBM (*N* = 58)	+ Pembrolizumab (MK-3475)	Phase IIB NCT02430363
AMG 386	GBM (*N* = 48)	+ Bevacizumab	Phase I/II NCT01290263
Vandetanib	GBM (*N* = 33)	+ Sirolimus	Phase I NCT00821080
